# Tunable Multifunctional Thermal Metamaterials: Manipulation of Local Heat Flux via Assembly of Unit-Cell Thermal Shifters

**DOI:** 10.1038/srep41000

**Published:** 2017-01-20

**Authors:** Gwanwoo Park, Sunggu Kang, Howon Lee, Wonjoon Choi

**Affiliations:** 1 School of Mechanical Engineering, Korea University, 145 Anam-ro, Seongbuk-gu, Seoul, 136-713, Republic of Korea; 2 Department of Mechanical and Aerospace Engineering, Rutgers University, 98 Brett RD, Piscataway, NJ, 08854, USA

## Abstract

Thermal metamaterials, designed by transformation thermodynamics are artificial structures that can actively control heat flux at a continuum scale. However, fabrication of them is very challenging because it requires a continuous change of thermal properties in materials, for one specific function. Herein, we introduce tunable thermal metamaterials that use the assembly of unit-cell thermal shifters for a remarkable enhancement in multifunctionality as well as manufacturability. Similar to the digitization of a two-dimensional image, designed thermal metamaterials by transformation thermodynamics are disassembled as unit-cells thermal shifters in tiny areas, representing discretized heat flux lines in local spots. The programmed-reassembly of thermal shifters inspired by LEGO enable the four significant functions of thermal metamaterials—shield, concentrator, diffuser, and rotator—in both simulation and experimental verification using finite element method and fabricated structures made from copper and PDMS. This work paves the way for overcoming the structural and functional limitations of thermal metamaterials.

Active control of thermal energy through mediums is a significant subject with applicability in diverse fields, from fundamental physics to practical applications. Understanding thermal energy transport at nano–microscales mainly depends on the phonon distribution and contact interface of layers^1^. Manipulation of thermal energy transport at macroscales has been regarded as equivalent to the control of heat flux through the material, because of its diffusive nature through specific mediums at continuum scales^1^,^2^. Most research about macroscale thermal transport has focused on the development of bulk materials or mediums that promote thermal transport with superior thermal conductivity or suppress heat transfer with thermal insulation and grain boundaries^3^,^4^. The progress of micro-nanotechnologies has enabled advanced research into a new class of materials with desirable properties, by means of using embedded fillers in composite structures^5^,^6^,^7^. However, such methods have reached the limit for achieving breakthroughs in terms of active control of thermal energy near local spots in macroscales, since they inevitably depend on thermal properties of mediums.

The development of metamaterials that are able to manipulate diverse physical properties using artificially designed structures have been introduced as a new approach to overcome previous limitations of transport phenomena through the mediums. Transformation optics was one of general approaches to design cloaking devices or optical waveguides^8^,^9^,^10^,^11^,^12^,^13^,^14^. This method was applicable to microwave frequencies as well^13^, and experimental verifications have been conducted in the visible wavelength region^15^. Furthermore, transformation thermodynamics has been recently extended to design new kinds of thermal metamaterials^16^, which actively control heat flux through diverse mediums in millimeter to centimeter scales, dominated by continuum mechanics.

The majority of early studies on large-scale thermal metamaterials were about how transformation thermodynamics could be used to design functional structures^16^,^17^,^18^. The design method for cloaking heat flux in a local region was derived from transformation optics^9^, while thermodynamic cells harvesting heat energy were achievable by ordering materials having different thermal conductivities^10^. Meanwhile, the thermal metamaterials designed using transformation thermodynamics were experimentally evaluated to perform the manipulation of heat flux^2^,^19^,^20^,^21^. The functional thermal metamaterials such as shield, concentrator, and rotator were fabricated by overlapping copper and polyurethane as the materials having the high and low thermal conductivities^2^,^18^,^19^,^20^,^21^,^22^,^23^,^24^,^25^,^26^,^27^,^28^,^29^. Thermal cloaking structures for molding the flow of heat on the metal surface were introduced by the combination of copper and PDMS^19^. In addition, anisotropically arranging two materials such as epoxy-rubber or wood-stainless steel could vary thermal cloaking performance in an identical design^20^. The functional extension from thermal cloaking to thermal camouflage was achieved by positioning thermal scattering structures in front of the shielding structure^21^. More recently, two-dimensional invisible sensor which enabled the sensing function without blocking or interfering incoming signals was developed for multi-physical wave^30^. As an extension of their functionalities, dual-function metamaterials that control both heat flux and electric current have been introduced by overlapping two distinct mediums^26^, effective medium theory^25^, or fan-shaped structure^31^.

In addition to the functionalities, the important issue for thermal metamaterials is how the optimal structures for specific target applications can be designed and manufactured using the material processing of prevalent technologies. In particular, thermal shifters that guided heat fluxes along the anisotropic direction^11^,^32^ were investigated by simulations and experiments^11^,^32^,^33^,^34^,^35^,^36^. Multilayered and diagonally oriented composites of different thermal conductivities could generate thermal elements for a horizontal temperature gradient^11^, as well as the transient propagation of heat fluxes^32^. Inversely, based on the heat flux mapping, thermal shifters could provide the information for interfacial thermal conductivity between two layered materials^33^. For optimal design and extension of the availabilities, the use of active modules^37^ or uniquely designed structures^38^,^39^,^40^ have added new types of functions. Thermoelectric modules around the target-local area could actively change the surrounding temperatures for the adaptive cloaking in response to externally supplied heat fluxes^37^. The fan-shaped structures could simultaneously produce thermal cloaking as well as thermal concentrating^38^. The combination of sensu-shaped units could achieve multiple thermal metamaterials such as concentrator, focusing-resolving, and uniform heating^39^. The addition of a complementary layer between the cloak region and the object could assign the feeling of heat to the target object in inner structures, whereas thermal cloaking was enabled in the outside region^40^.

However, one distinct structure has been designed in order to implement one specific task as a thermal metamaterial. The fabrication for the individual function should be performed with the specifically optimized structures. For diverse functions, the fabrication of multiple numbers of designed thermal metamaterials would be inevitable. The above limitations for diverse functionalities cause the additional resources for a new design of the target function. Moreover, thermal metamaterials generally use curved lines or complex structures for the active guidance and continuous distortion of the heat flux. Fabrication of such complicated structures needs precise manufacturing equipment as well as high cost and lengthy processing time. Furthermore, although attempts using various additives or new materials have advanced the functions, the basic shape of thermal metamaterials has been restricted to the cylindrical shape.

In this work, we firstly report on tunable thermal metamaterials using assembly of unit-cell thermal shifters in order to enhance their multifunctionalities as well as manufacturabilities. To date, thermal metamaterials have been designed in continuous structures for making the designed heat fluxes depending on their functions at the macroscale. We introduce thermal metamaterials using the assembly of discretized heat flux lines, inspired by the digitization of a two-dimensional (2D) image. The continuous heat flux in thermal metamaterials can be separated as disassembled unit-cells in tiny areas, representing the individually discretized heat flux lines in local spots. The each unit-cell is made of one thermal shifter, which is known as the simplest structure for guiding heat flux along the one specific direction^11^. Manufacturing thermal shifters is relatively simple because the basic structures are composed of an alignment of straight lines of two materials possessing different thermal conductivities, while the differently aligned angle converts the direction of the discretized heat flux^32^. Furthermore, the diversely programmed-recombinations of thermal shifters in tiny areas, similar to LEGO blocks, enable the multifunctional thermal metamaterials based only on the re-assembly of unit-cells (Fig. 1). This study discretizes and redesigns the four representative thermal metamaterials (shield, concentrator, diffuser, and rotator) as multiple unit-cells of 10 by 10 thermal shifters (a total 100 units). The optimized alignment of unit-cells can regenerate the representative functions with the identical thermal shifters. For a demonstration of the designed structures, 4 by 4 thermal shifters with differently aligned angles (15°, 45°, 75°, totally 16 unit-cells) are fabricated and differently assembled to confirm multi-functions as thermal shield, concentrator, diffuser, and rotator. The developed tunable multifunctional thermal metamaterials can be utilized in further enhancement of manufacturability and reusability because they can achieve diverse functions with only a change in alignment.

## Results

### Design Principles of Tunable Multifunctional Thermal Metamaterials

**Figure 2:**
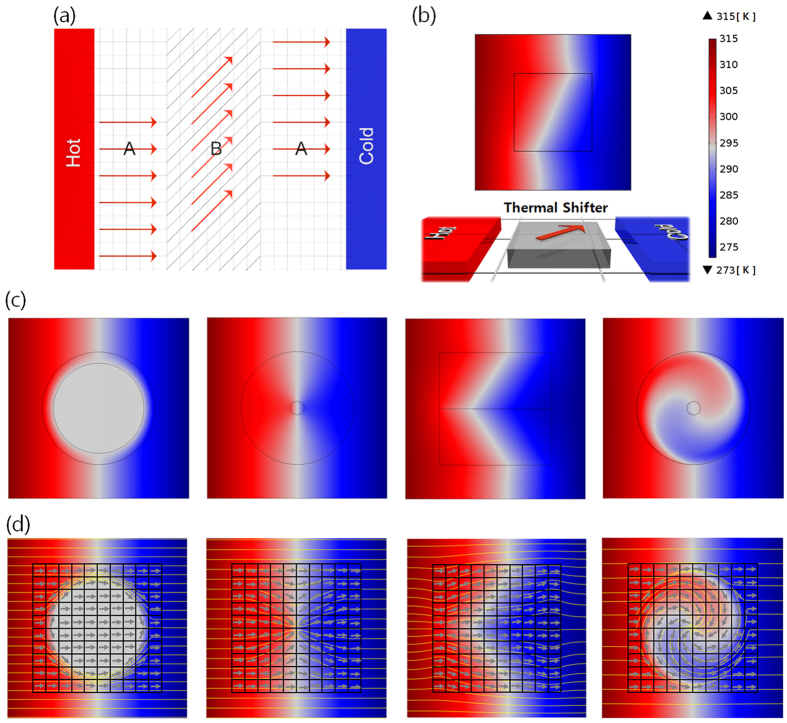
Design principles of tunable multifunctional thermal metamaterials. (**a**) Sketch of thermal conductivity distribution in the normal Cartesian coordinate region A and the transformed coordinate system B for thermal shifters. The 45° tilted angle in region B induces the bending of thermal transport. (**b**) Temperature profile of a thermal shifters in 45° tilted angle, between the hot and cold sides. Target functions of thermal metamaterials - shifter, concentrator, diffuser and rotator from left to right sides. (**c**) Designs of representative functions based on transformation thermodynamics and (**d**) decomposition of heat flux and thermal conductivity as unit-cells in local areas. The yellow lines indicate the heat flow directions, which are originated from the simulated results for four different functions. The gray arrows indicate the guiding directions by thermal shifters to realize the identical functions with the conventional thermal metamaterials.

Basic structures of thermal shifters as unit-cells are designed to guide heat flux along the desired direction by transformation thermodynamics (see ‘Transformation thermodynamics’ in Methods section). Fundamentally, tensors are general objects for expressing transformed coordinates since their mathematical notation can independently describe any kind of coordination system, regardless of its original coordinates. The heat conduction equations are applicable regardless of the specific coordinate system, as well. The transformation between the original coordinate space and the transformed coordinate space can derive the design of thermal shifters, as shown in Fig. 2 (see ‘Design method for thermal shifters’ in Methods section). Region A in Fig. 2a shows the original coordinate space, whereas region B indicates the transformed coordinate space, which is tilted by *θ°* for guiding the heat flux along the specific direction. The above transformed thermal conductivity formulates the manipulation of the discretized heat flux. As a representative example of thermal shifter design for guiding heat flux, a 45° tilted angle of thermal conductivity (*θ* = 45°) was assumed, and the corresponding temperature profile was analyzed by finite element method (FEM) using COMSOL^®^ Multiphysics. As shown in Fig. 2b, it is verified that tilting thermal conductivity along the specific direction can effectively bend the heat flux between low temperature and high temperature reservoirs. The bending degree of temperature gradient would depend on the tilted angle of thermal conductivity inside the thermal shifters.

Thermal metamaterials for representative functions, such as shield, concentrator, diffuser, and rotator were redesigned for the local manipulation of heat flux and intended thermal energy distribution, based on transformation thermodynamics (Fig. 2c). According to the target application, they all have optimal-continuous heat flux lines, which are confined in the specific direction, by means of mismatching thermal conductivities in local areas. This common characteristic indicates that thermal metamaterials contain a lot of information about the direction of heat fluxes, and they are made from the assembly of infinitely large numbers of straight heat flux lines. Such continuous heat fluxes in local areas can be approximated to the assembled tiny unit-cells having straightly confined heat flux lines that are implemented by thermal shifters. The optimally discretized heat flux lines are extracted by the first derivatives in continuous distribution of thermal conductivities and temperature profiles (Fig. 2d), which were formulated in thermal metamaterials. The individual heat flux is converted to the tilted angle of one unit-cell thermal shifter. Generally, a critical barrier for the further development of thermal metamaterials is the complex design and the difficulty of the fabrication for specific functions. The yellow lines in Fig. 2d indicate the heat flow directions, which are originated from the simulated results for four different functions. Those continuous lines are divided as discretized-straight lines which can be realized in the designed assembly of unit-cell thermal shifters, because one thermal shifter can confine the heat flow and conduct the linear guidance of thermal energy. The gray arrows indicate the guiding directions by thermal shifters to realize the identical functions with the conventional thermal metamaterials. As shown in Fig. 2d, such alternative design using thermal shifters, the simplest structures for guiding heat flux, is applicable to the digitization of two-dimensional heat flux maps. The accuracy of regeneration of heat flux maps would depend on the resolutions that are equivalent to the number and size of unit-cell thermal shifters. Large thermal shifters (meaning low resolution) are easy for manufacturing, however with a loss of accuracy. On the other hand, the assembly of small thermal shifters (indicating high resolution) is relatively difficult for manufacturing, but has more accurate regeneration of designed heat flux maps.

**Figure 3:**
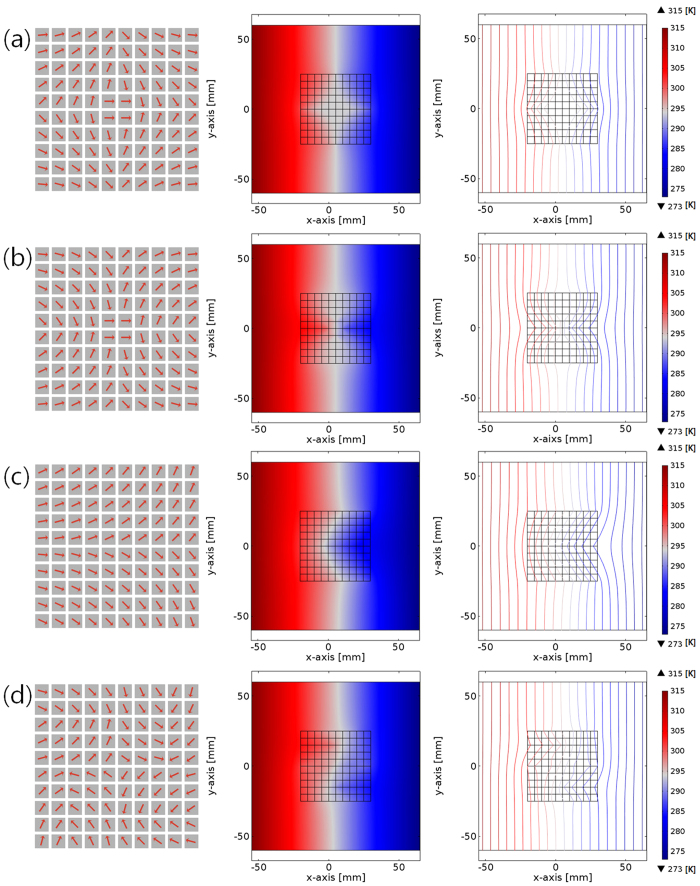
Assembly of unit-cell thermal shifters for diverse functions of thermal metamaterials. Designed assemblies of thermal shifters (left), temperature profiles (middle), and isothermal plots (right) of tunable thermal metamaterials between hot and cold sides, corresponding to (**a**) shield, (**b**) concentrator, (**c**) diffuser and (**d**) rotator.

The designed assembly of 100 (10 by 10) unit-cell thermal shifters has successfully formulated four different thermal metamaterials (shield, concentrator, diffuser and rotator) (Fig. 3). The pre-design of heat flux maps and the corresponding thermal conductivities were carried out by transformation thermodynamics, as described in Fig. 2c and d, while the analysis for temperature distribution was conducted by FEM. The dimension of assembled structures for one function was 5 cm × 5 cm, while the unit-cell size of the individual shifter was 5 mm × 5 mm. For the implementation of the specific thermal function, 100 thermal shifters, rotated in increments of 5° between 0° and 90° were differently assembled for local manipulation of directionally discretized heat flux in tiny unit-cells, as previously designed distribution of thermal conductivity.

The assembly designs of thermal shifters for thermal shield, concentrators, diffuser, and rotator are based on the specific arrangements of four mid-size components that were fabricated by the assembly of 25 thermal shifters, as shown in the assembled design in Fig. 3a,b,c and d, respectively. The design purpose of the thermal shield and concentrator is the realization of thermally shielded and concentrated regions at the point of the intersection of four mid-size components. On the other hand, the thermal diffuser and rotator should generate a wide thermal-diffusion and an inverted temperature gradient compared to the original direction of heat flux at the point of the intersection of the four mid-size components. The heat flux through the assembled thermal metamaterial is induced by the fixed temperature gradient on the left side (hot side: 315 K) and the right side (cold side: 273 K), as shown in Fig. 3. As a first step for assembly designs, the first derivatives of the ideal thermal conductivity distribution are calculated from thermal metamaterials in the continuous-cylindrical structures (shield, concentrator, diffuser, rotator, respectively). Next, in the simultaneous consideration of the predetermined unit-cell size (one thermal shifter) and the extracted first derivatives, the inclined angles of thermal shifters at the specific spots are decided; they represent the slopes of discrete heat flux lines inside the assembled structures (see Methods section for the detailed assembled designs of shield, concentrator, diffuser and rotator). As shown in the temperature profiles in middles of Fig. 3a and b, the symmetric combinations of 4 mid-size components (25 thermal shifters, respectively) formed the shielded area and the concentrated area in the heat flux between the hot and cold sides. Meanwhile, the asymmetrical arrangements of 100 thermal shifters in specific regularities formed the rapidly diffusive heat flux from the center position (middle of Fig. 3c) or the inversion of temperature gradient at the intersection (middle of Fig. 3d), when the hot and cold temperature reservoirs were placed at the left and right sides. The external area outside thermal metamaterials had the high thermal conductivity from the conductive component, while unit-cell thermal shifters realized the designed thermal conductivity, *k*′ which were implemented by the layered structures of the conductive and non-conductive materials in high and low thermal conductivities.

The isothermal plots of functions can confirm the realization of thermal metamaterials using the assembly of thermal shifters. The empty spot of the isothermal line of the temperature distribution from the thermal shield proves that temperature gradient does not exist in the center area (right of Fig. 3a), whereas, the dense spot of the isothermal lines from thermal concentrator shows that thermal energy is focused on the center due to a large temperature gradient in the narrow regime at the center (right of Fig. 3b). The diagonal alignments of the isothermal lines at the top and bottom near the center position of thermal diffuser confirmed that a drastic thermal diffusion was derived in the design (right of Fig. 3c). Finally, the reverse alignment of isothermal lines from the thermal rotator indicated that there was strong distortion of heat flux near the center position (right of Fig. 3d). Furthermore, such analysis indicates that the different assemblies of the identical thermal shifters (10 by 10 shifters), assembled by the programmed combination of discretized heat flux lines can perform multi-functions such as shield, concentrator, diffuser, and rotator. It should be noted that identical thermal shifters were used to perform four different functions, and they were only rearranged for each functions. The discretized heat flux lines can potentially form any kind of thermally functional local manipulation through a rearrangement of assemblies in the identical thermal shifters. The temperature distortion in the external regime occurred to cancel out the temperature mismatch at the interface of inner thermal metamaterials and the external environments. Such distortion can be reduced by changing the properties of materials or the recombination of material choices.

### Design Optimization for Fabrication of Tunable Multifunctional Thermal Metamaterials: Contact Interfaces and Reduction of Numbers of Unit-Cells

**Figure 4:**
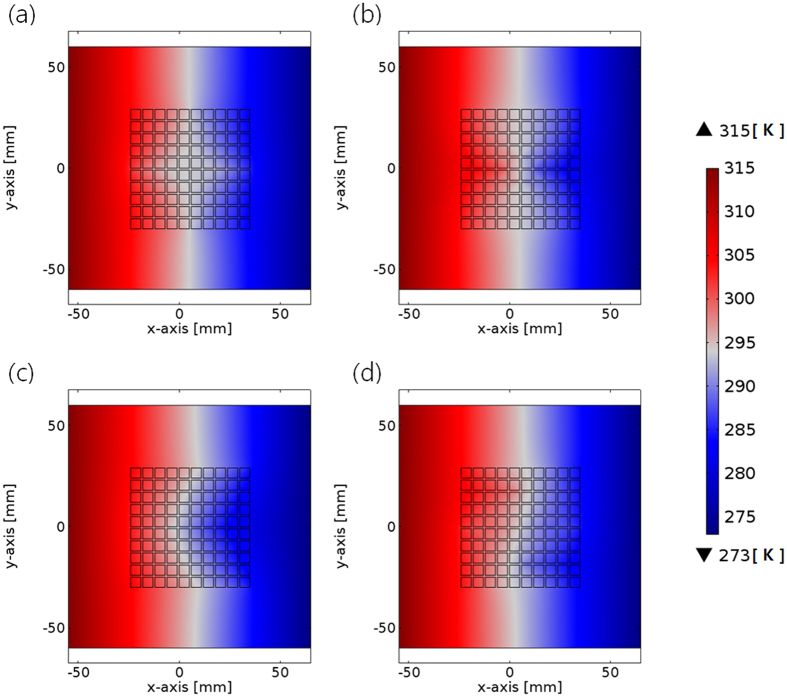
Temperature profiles of multi-functional thermal metamaterials, which are composed of the assembly of 100 unit-cell thermal shifters with the consideration of the contact interfaces (~2 mm). (**a**) Thermal shield, (**b**) thermal concentrator, (**c**) thermal diffuser, and (**d**) thermal rotator.

For the fabrication of thermal metamaterials using the assembly concept, the contact interface between thermal shifters should be considered as a design factor, because the resistance at the interface is inevitable for the connection of the individual unit-cells. In the previous designs, it is assumed that discretized heat flux in one thermal shifter was naturally transferred to the neighboring thermal shifters without thermal dissipation. However, in reality, the contact interface around a thermal shifter is mandatory to maintain its original shape and to transfer thermal energy to other unit-cells. In this condition, the thermal contact interface can interrupt the pre-designed discretized heat flux and degrade the performance. Therefore, the overall performance of thermal metamaterials using unit-cells thermal shifters was reevaluated with the consideration of the contact interfaces between thermal shifters, as shown in Fig. 4. All boundary conditions and thermal reservoirs for hot and cold sides (315 K and 273 K) were identical to the previous simulations, except for the insertion of the contact interfaces. With the moderate thickness of the contact interfaces (~2 mm), the multiple functions such as shield (Fig. 4a), concentrator (Fig. 4b), diffuser (Fig. 4c), and rotator (Fig. 4d) worked effectively, although there was a little thermal degradation due to the distortions of temperature gradient near the boundaries. However, the thicker the contact interfaces, the more distorted the temperature profiles were observed, with significant loss of functions. It turns out that the fabrication of thermal metamaterials using the LEGO assembly of unit-cell thermal shifters would be feasible if thin contact interfaces surround the individual thermal shifters. Contact interfaces thicker than the critical value greatly disturb the discretized heat fluxes between thermal shifters. The minimum thickness of the contact interfaces that is able to maintain the rigidity and shape of thermal shifters should be used for the fabrication. The resolution of the contact interfaces is also limited by the method of its fabrication.

**Figure 5:**
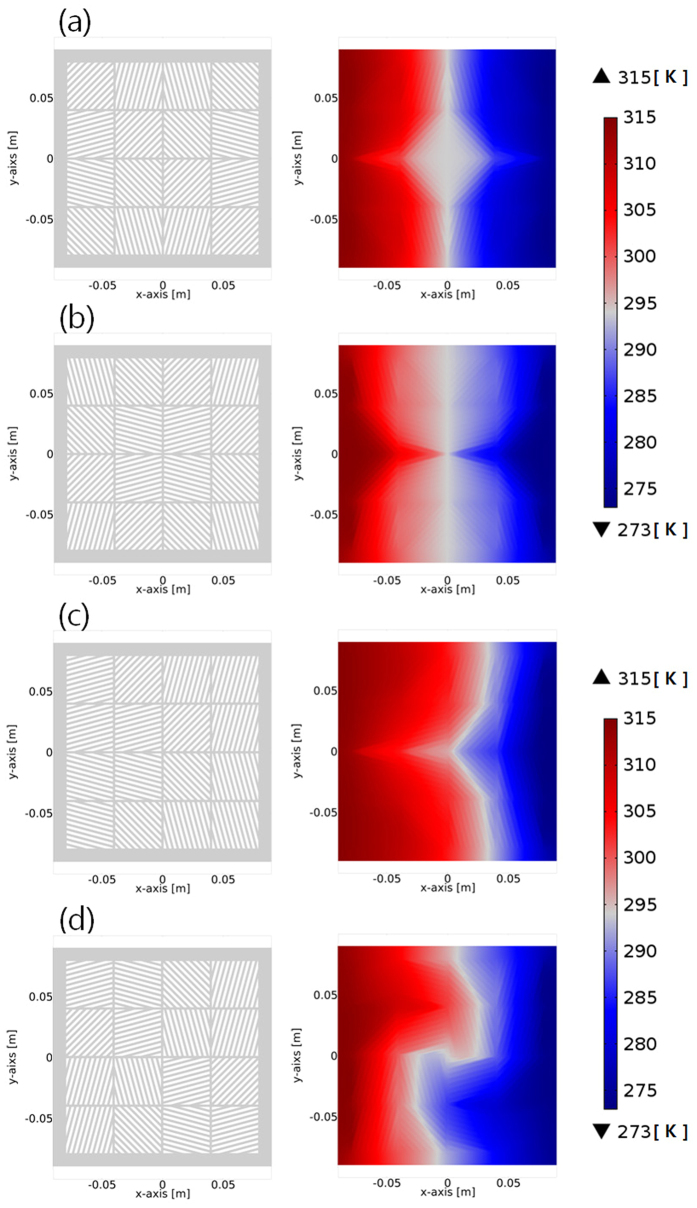
Design and performance of tunable multifunctional structures in reduced numbers of unit-cell thermal shifters. Assembled designs in 4-by-4 thermal shifters (left) and corresponding temperature profiles (right) for (**a**) thermal shield, (**b**) thermal concentrator, (**c**) thermal diffuser, and (**d**) thermal rotator.

In order to confirm the performances of thermal metamaterials using the assembly of thermal shifters, the fabrication of the thermal metamaterials with reduced numbers of unit-cell thermal shifters would be effective because of its manufacturability and easily assembled structures from the identical thermal shifters (Fig. 5). Here, a total of 100 thermal shifters (10 by 10) in the previous modeling, which are composed of inclined angles from ±0° to ±90° with a ±5° increase rate, is reduced to 16 thermal shifters (4 by 4), which are composed of thermal shifters in ±15°, ±45°, ±75° inclined angles (left of Fig. 5a–d). The contact interface as described in the previous part was considered as the additional design factor. It should be highlighted that the different assemblies of the identical thermal shifters (4 by 4, total 16 unit-cells) can still provide the multifunctionality, shield (Fig. 5a), concentrator (Fig. 5b), diffuser (Fig. 5c), and rotator (Fig. 5d). All 4 by 4 assemblies showed a little change of the temperature distribution, while the individual function was maintained, in comparison with a 10 by 10 assembly. Due to the decrease of the total number of unit-cell thermal shifters, the isothermal lines in temperature gradient revealed a more discontinuous trend (right of Fig. 5a–d). Similar to the pixels of images, the larger pixels, which are composed of unit-cell thermal shifters, inevitably make the more discontinuous isothermal lines. It means that the identical functions, such as shield, concentrator, diffuser, and rotator are still feasible with a smaller number of thermal shifters than that of the previous modeling, while the curvature of isothermal lines are highly dominated by the specific size of unit-cell thermal shifters.

### Experimental Verification of Tunable Multifunctional Thermal Metamaterials

**Figure 6:**
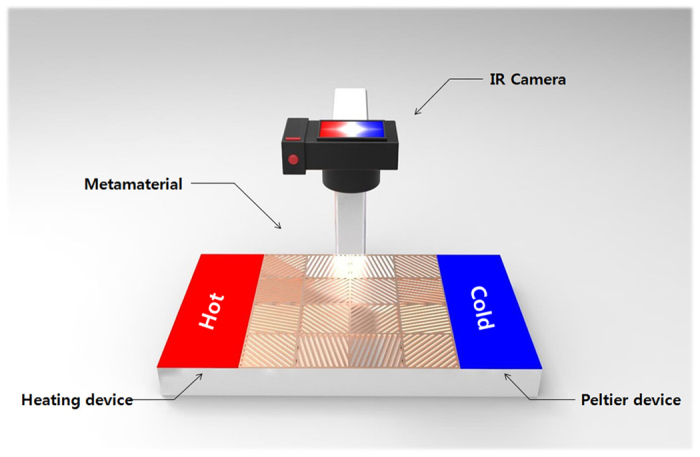
Scheme of the experimental setup to evaluate the performances of thermal metamaterials. Two thermoelectric devices (heating and Peltier modules) are fixed to maintain a temperature gradient at left and right sides (308 K and 288 K), while thermal IR camera captures the temperature profile of a target object in real-time.

The experimental setup which were composed of thermal infrared camera, heating-cooling devices, and fixtures, was prepared to evaluate the performances of tunable multifunctional thermal metamaterials (Fig. 6). In the setup, it is able to form the hot (high temperature boundary) and cold (low temperature boundary) sides to the edges of assembled structures, and measure the real time change of temperature distribution (See Methods section for details).

**Figure 7:**
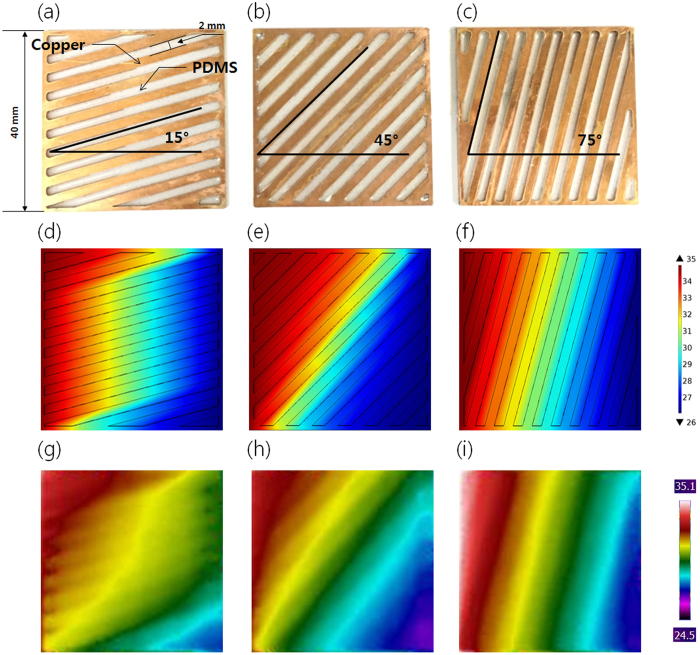
Fabricated thermal shifters, and performance evaluation. Thermal shifters are made by copper and PDMS, in (**a**) 15°, (**b**) 45°, and (**c**) 75° inclined principle axis. Simulation results of temperature profiles for fabricated structures, using FEM in (**d**) 15°, (**e**) 45°, and (**f**) 75° inclined principle axis. Experimental verification of temperature distribution of fabricated thermal shifters using thermal IR camera in (**g**) 15°, (**h**) 45°, and (**i**) 75° inclined principle axis.

The overall thermal conductivity for thermal metamaterials is provided as tensor properties, not scalar properties. The fabrication of perfectly matched structures using tensor properties is quite challenging, and a diverse method such as the effective medium theory has been applied to the manufacturing process for the target structures^25^,^26^. However, in general, the functions of thermal metamaterial have been achieved by layering structures of high thermal conductivity materials and low thermal conductivity materials. The high thermal conductivity materials are mainly in charge of transferring thermal energy and constructing the heat flux lines, whereas the low thermal conductivity materials confine thermal energy and guide the heat flux lines to the intended directions. These layered structures enable simpler, rather than complex structures, with continuous change of thermal conductivity. The unit-cell thermal shifters in the multifunctional thermal metamaterials are composed of layered, two-dimensional structures of copper as the high thermal conductivity material (*k* = 400 W/m·K) and PDMS (Polydimethylsiloxane) as the low conductivity material (*k* = 0.2 W/m·K) (Fig. 7). The three different thermal shifters in 15°, 45°, and 75° inclined angles are designed and fabricated as the basic unit-cells (total 16 thermal shifters) (Fig. 7a,b and c). As it is confirmed in the previous design of 4-by-4 assembly with contact interfaces (Fig. 5), only three types of unit-cells (15°, 45°, and 75°) are enough to give the four representative multifunctions using the identical thermal shifters via different assemblies. Laser copper etching and continuous filling of PDMS were used to fabricate one unit-cell in the designed dimension (40 mm × 40 mm in area and 1 mm in thickness for one thermal shifter). In this fabrication, the pattern slits for each layer were about 2 mm for both the inclined line of copper and the gap for filling PDMS layers. The 15°, 45°, and 75° inclined thermal shifters should form the differently inclined heat flux lines and isothermal lines between the hot (~308 K) and cold (~298 K) sides, as described in the simulation results, respectively (Fig. 7d–f). As expected, the fabricated thermal shifters with copper and PDMS showed nearly the same performance for the manipulation of heat flux along the 15°, 45°, and 75° inclined angles at the steady state when the thermal IR camera measured the real-time temperature profiles (Fig. 7g–i).

**Figure 8:**
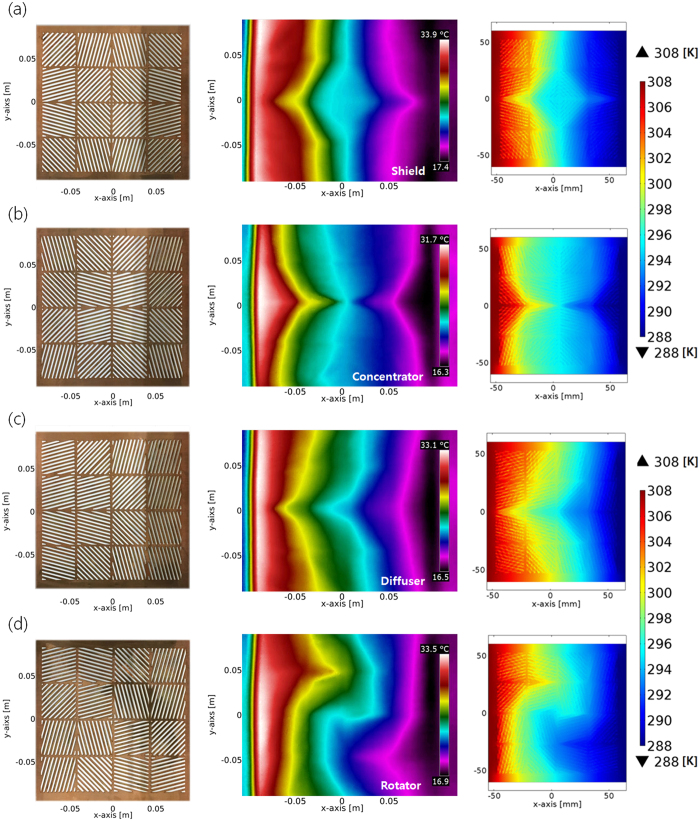
Fabrication of tunable multi-functional thermal metamaterials and manipulation of local heat flux in corresponding assemblies. Fabricated assembly of identical thermal shifters (16 unit-cells) for different functions of thermal metamaterials (left), the manipulated temperature profiles (middle) and the simulation results considering the convection (right) of (**a**) thermal shield, (**b**) thermal concentrator, (**c**) thermal diffuser, and (**d**) thermal rotator. Ambient temperature 293 K, h = 10[W/m^2^K] for convection analysis.

The tunable thermal metamaterials by the diverse assemblies of 4-by-4 unit-cell thermal shifters in ±15°, ±45°, and ±75° principle directions were fabricated by the laser etching of copper and continuous filling procedure of PDMS (Fig. 8). As it was designed in Fig. 5, the thermal shield, concentrator, diffuser and rotator consisted of the 16 thermal shifters (40 mm × 40 mm in area), and one assembled metamaterials was a square shape (16 cm × 16 cm in area). In addition, the copper interfacial layers (~2 mm) for linking the thermal shifters were inserted into the boundary between the differently inclined thermal shifters, because the effectiveness of contact interfaces was evaluated in the simulation.

The multifunctionality of thermal metamaterials using the assembly of identical unit-cell thermal shifters appears in the temperature profiles as measured by thermal IR camera (Fig. 8). First, the thermal shield (Fig. 8a) forms a diamond-shape that has no temperature gradient in the central area, when the temperature gradient from hot and cold sides is applied. Four thermal shifters in the ±45° principle direction surround the target shielding regime, and other twelve thermal shifters in the ±15°, ±45°, and ±75° principle directions enclose the four inner thermal shifters (left of Fig. 8a). If the specific object needs protection from the rapid temperature change in the local area, the manipulation of heat flux using the assembly of thermal shifters that surrounds the target object would be applicable. Thus, the design of the thermal shield from the digitized structures provides an efficient way to prevent the thermal damage in the certain area, as shown in Fig. 8a. The precision instrument should maintain high accuracy during the manufacturing process, or the operating condition, while the sudden increase of temperature in the operation generally causes structural distortion due to thermal expansion. In this case, the shield of thermal gradient is mandatory for vital and sensitive components. The addition of simple-digitized structures of thermal shifters would help to protect such structural distortion and improve the stability.

The second function, thermal concentrator, can be achieved through a different assembly of the identical unit-cell thermal shifters (Fig. 8b). Four thermal shifters in the ±15° principle direction surround the target shielding regime, and other twelve thermal shifters in the ±15°, ±45°, and ±75° principle directions enclose the four thermal shifters (left of Fig. 8b). This assembly of unit-cell thermal shifters significantly narrows the temperature gradient in the central regime. The spacing between isothermal lines near the central area is lesser than it is in the other regions. This indicates that heat is efficiently collected to a specific target area with the maximized temperature gradient in the narrow regime from the limited source of thermal energy. This design would be valuable for improving the functionality of materials in extreme environments. For example, specific functions or properties of materials are quite sensitive to temperature. Sometimes, they lose the usability in low temperatures. A thermal concentrator using unit-cell thermal shifters can provide the concentration to the sensitive material when there are scattered high temperature sources such as the human body, or thermal energy from the frictional components. The addition of simple-digitized structures of a thermal concentrator would help to collect the scattered thermal energy for the target object.

The third function, thermal diffuser, can be also achieved in the new assembly of identical unit-cell thermal shifters (Fig. 8c). From the left side at a hot temperature, four thermal shifters are placed in the ±15° principle direction, and more inclined thermal shifters are aligned to the right side at the colder temperature. In details, the four thermal shifters in the ±15° and ±45° principle directions, four thermal shifters in the ±45°, and ±75° principle directions, and four thermal shifters in the ±75° principle direction are fixed from left to right sides (left of Fig. 8c). In this function, the heat flux spreads out to both side-ends from the left (hot temperature) to the right (cold temperature) directions. This indicates that the effective thermal dissipation from the central position in the horizontal direction is achieved. The function of a thermal diffuser is useful to dissipate and remove the redundant thermal energy from the central position in the horizontal direction. It provides a very useful approach to lower the temperature in the target area when the target object is exposed to high temperature sources on one side, or a point-heat source. In this environment, a thermal diffuser broadly distributes the heat energy from the thermal energy source to the outside.

The last function, thermal rotator, is completed by the spiral assembly of unit-cell thermal shifters (Fig. 8d). In particular, the four thermal shifters in the ±15° and ±75° principle directions are assembled near the central regime, while twelve thermal shifters in the ±15°, ±45°, and ±75° directions spirally enclose the inner structures (left of Fig. 8d). In this function, the inversion of hot and cold sides in the central regime is observed (right of Fig. 8d). The temperature gradient from right to left is hot to cold near the target area, whereas the intrinsic direction from the hot side to cold side is left to right. In comparison with the general properties of materials, it is a unique feature to make the reverse temperature gradient with the original direction of thermal energy distribution, because thermal energy always flows from the hot side to the cold side.

## Discussion

Supplementary Figure 1 summarizes the multifunction features of the assembly of unit-cell thermal shifters. Due to the layer structures of copper and PDMS, there is a discretized line of high thermal conductivity, and one of low thermal conductivity. Furthermore, the boundary between the individual thermal shifters was observed due to the integration of unit-cells, in comparison with the other thermal metamaterials. Although the boundary effect between unit-cell thermal shifters is inevitable, the assembly concepts are still effective to form the specific functions of thermal metamaterials. In addition, identical thermal shifters can differently manipulate the heat flux in the diverse assembly design.

The choice of a specific material for high thermal conductivity layer is a crucial factor to determine the overall performances of thermal metamaterials in assembly designs (See Supplementary Figure 2). When stainless steel (*k* = 16 W/m·K) was used as the high thermal conductivity layer, the reassembly of thermal shifters was not able to show the functions as thermal metamaterials. On the other hand, the 5Al bronze (*k* = 80 W/m·K) moderately accomplished the specific functions, even though its temperature profile was more dissipated than that of a copper case in Fig. 8. The experimental verification based on stainless steel cases confirms that the lower thermal conductivity in the main layer for heat transfer causes the more dissipation of thermal energy in designed structures, and results in the loss of functions (See Supplementary Figure 3).

Cloaking is one of the most important function for thermal metamaterials, although tunable thermal metamaterials have focused on 4 different functions. In this respect, the perturbations of temperature in external area is interesting observations, when tunable thermal metamaterials are embedded in bigger hosts. The assembled models for shield, concentrator, diffuser and rotator were placed in bigger hosts (*k* = 400 W/m·K) and the overall temperature profiles were simulated in Supplementary Figure 4. The significant perturbations of temperature profiles in the bigger hosts were not observed, although there was a little change near the interfaces. Temperature profiles of bigger hosts having thermal shifters are shown in Supplementary Figure 5. Similar to tunable thermal metamaterials, a little perturbation of temperature gradient was observed, while the overall aspect depended on the inclined angles of thermal shifters. The perturbation of temperature profiles is highly correlated to the thermal conductivity of bigger hosts. The simulated results in different thermal conductivities of bigger hosts were shown in Supplementary Figure 6 to describe the changes of perturbation, while the assembled unit-cell thermal shifters enabled four different thermal functions. Thermal conductivities of components for functional areas were fixed (copper: *k* = 400 W/m·K, PDMS: *k* = 0.2 W/m·K), whereas the thermal conductivities of the bigger hosts were changed from 400 W/m·K to 100 W/m·K to 40 W/m·K (left, middle, and right in Supplementary Figure 6). As the thermal conductivity of the bigger host decreased, the perturbation of temperature profile gradually disappeared. However, too low thermal conductivity of the bigger host induced the performance deterioration of thermal functions. Therefore, the further optimization of thermal conductivities for hosts and metamaterials would be needed to include the cloaking function.

## Conclusion

In summary, we propose the design and experimental verification of tunable multifunctional thermal metamaterials using the designed assembly of unit-cell thermal shifters. The diverse combinations of thermal shifters as unit-cells could individually manipulate the local heat flux for specific functions of a thermal shield, concentrator, diffuser, and rotator. This newly developed concept for assembled design of thermal metamaterials facilitated the fabrication of simple structures using unit-cells, which overcame the structural limitations of conventional thermal metamaterials. The unique approach in tunable multifunctional thermal metamaterials is applicable for improving scalability and manufacturability. The fabrication of unit-cells such as thermal shifters is relatively easy, and it extends the flexibility and the applicability of thermal metamaterials at the macroscale. Furthermore, this concept would contribute to industry or military applications where components might need the manipulation of the local heat flux. In particular, electronic products, military supplies, and high-precision machine parts are usually susceptible to thermal damage. According to the application of multifunctional thermal metamaterials composed of unit-cells, thermal energy through conduction, convection, and radiation can be effectively managed, and it may revolutionize the way various products are designed using enhanced thermal energy controls. For examples, the military drone may be one of the potential applications using the assembly of unit-cells for thermal metamaterials. The military drone is generally exposed to the extreme temperature change (hot and cold weather), and they should maintain the uniform functions, such as power sources and optical parts, while the lightweight is quite important to improve an efficiency of execution of operations. In this respect, the assembly of unit-cells thermal components can be effectively applied to the functional parts for the protection of thermal damages. Furthermore, the lightweight would be achievable, simultaneously, because it uses the metal and polymer layered structures. Lastly, the results of this study could help in extending the manufacturing method for thermal metamaterials with scalability. Thermal metamaterials can be digitized by small unit-cells, which provide manufacturable metastructures in existing material processing. One of their combinations will complete the one specific function of thermal metamaterials, while the change of the assembly will assign multifunctional roles in same unit-cells.

## Methods

### Transformation thermodynamics

The originally defined coordinate system 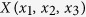 is converted to a tensor expression in a transformed space, 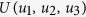 which means a coordinate transformation. In transformation thermodynamics, the heat conduction equation as described by a tensor expression is


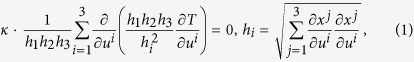


where *κ* is the thermal conductivity, *T* is the temperature, *x* is the coordinate of the original system, and *u* is the coordinate system in the transformed space. The tensor expression can represent the heat conduction equations in various coordinate systems such as Cartesian, cylindrical, spherical, and the specifically transformed coordinates. Therefore, the physical characteristics of the heat conduction equation in the original system 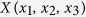 are preserved in its tensor expression, 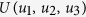 in transformed space. Transformation thermodynamics, based on this coordinate transformation allows for the derivation of transformed thermal conductivity, expressed as


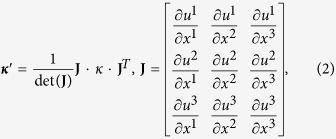


where **J** is the Jacobian of the coordinate transformation, the superscript ^*T*^ indicates the transposed matrix, and det(J) is the determinant of the Jacobian matrix. It should be noted that ***κ***′ is the transformed thermal conductivity of the coordinate system, which is derived from the 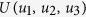 in the transformed space, and **J** represents the correlation between the 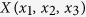 in the original coordinate space and the 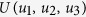 in the transformed coordinate space. The inverse transformation provides the information of the thermal conductivity in the original Cartesian coordinate space, 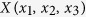, from the thermal distribution in a tensor expression, 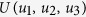. Applying the transformed thermal conductivity in the original coordinate space (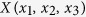) results in the correlated heat conduction and temperature distribution in the transformed coordinate space (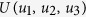), and vice versa, because the transformed thermal conductivity *κ*′ is correlated with the general coordinate system by linking the Jacobian of the coordinate transformation, **J** matrix.

### Design method for thermal shifters

Region A in Fig. 2a shows the original coordinate space, whereas region B indicates the transformed coordinate space, which is tilted by *θ°* or guiding the heat flux along the specific direction. The mathematical formula for the relation between region A and region B is


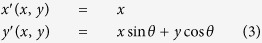


where superscript ′ indicates the transformed coordinate space. The Jacobian matrix of the transformation from region A to region B is


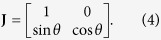


Finally, converting *κ* to ***κ***′ using the Jacobian relation, the transformed thermal conductivity is


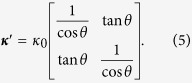


### Assembled design of thermal shield

For the thermal shield (left of Fig. 3a), in the left-top mid-size component (25 unit-cells), the angle of the thermal shifter at the left-top corner was 5°; the inclined angle incrementally deviated from the left-top corner up to 75°, periodically. The right-top and left-bottom mid-size components were symmetrical structures of the left-top component with respect to the y-axis and x-axis, respectively, whereas the right-bottom mid-size component was symmetric with respect to diagonal axis. The more the heat flux approached the point of intersection of the four mid-size components, the more the thermal gradient disappeared.

### Assembled design of thermal concentrator

In the design of thermal concentrator (left of Fig. 3b), the left-top midsize component (25 unit-cells) had the −5° inclined angle of thermal shifter at the left-top corner; the inclined angle incrementally decreased from the left-top corner up to −75°, for the negative direction of heat flux. The symmetric relation between the four mid-size components was identical to a thermal shield case. The more the heat flux approached the center of the mid-size components, the sharper the thermal gradient appeared.

### Assembled design of thermal diffuser

For the thermal diffuser (Fig. 3c), the two mid-size components at the left-top and left-bottom should lead the moderate change of the heat flux to the orthogonal direction, whereas the other two mid-size components at the right-top and right-bottom should induce the drastic change to the orthogonal direction. Moving from left to right inside the assembly of thermal shifters, the inclined angle gradually increased from 5° to 75° in the left-top and right-top mid-size components, and decreased from −5° to −75° in the left-bottom and right-bottom mid-size components (left of Fig. 3c). In other words, the top mid-size components and the bottom mid-size components were symmetric with respect to the x-axis. The wide distribution of thermal energy distribution suddenly appeared near the intersection of four mid-size components.

### Assembled design of thermal rotator

A thermal rotator (Fig. 3d) should produce the strong spiral heat flux inside each mid-size component. For this purpose, each mid-size component was composed of two parts for the formation of spiral heat flux; one half mid-size component with 15 shifters for the main distortion of heat flux and the other half mid-size component with 10 shifters for the curvature of the distorted heat flux. Actually, the half mid-size component with 15 shifters and 10 shifters showed the identical design with the mid-size component in thermal concentrator, except for size. The one in a 5-by-3 arrangement (15 shifters) was opposite to the other one in a 5-by-2 arrangement. The formation of a face-to-face arrangement using non-identical half mid-size components enabled the programmably distorted thermal concentration along the mid-size components at the left-top, right-top, right-bottom, and left-bottom. The sequential accumulation of the asymmetric heat flux, which was derived from the combination of 100 thermal shifters, emerged as the strongly distorted spiral heat flux at from the center position between the four mid-size components.

### Experimental set-up for performances evaluation of thermal metamaterials

A thermal IR (infrared) camera (FLIR T-420) is placed on the top of the target object to capture the temperature distribution of thermal metamaterials, while two thermoelectric devices (heating and Peltier modules) are used to maintain the hot (~308 K) and cold (~288 K) temperatures at the left and right sides (Fig. 6), respectively. As soon as the operation of thermoelectric devices commences, a transient heat transfer occurs through thermal metamaterials from the left to the right sides. In a few seconds, the temperature distribution reaches the steady state, which is comparable to the design and simulation results.

## Additional Information

**How to cite this article**: Park, G. *et al*. Tunable Multifunctional Thermal Metamaterials: Manipulation of Local Heat Flux via Assembly of Unit-Cell Thermal Shifters. *Sci. Rep.* **7**, 41000; doi: 10.1038/srep41000 (2017).

**Publisher's note:** Springer Nature remains neutral with regard to jurisdictional claims in published maps and institutional affiliations.

## Supplementary Material

Supplementary Information

## Figures and Tables

**Figure 1:**
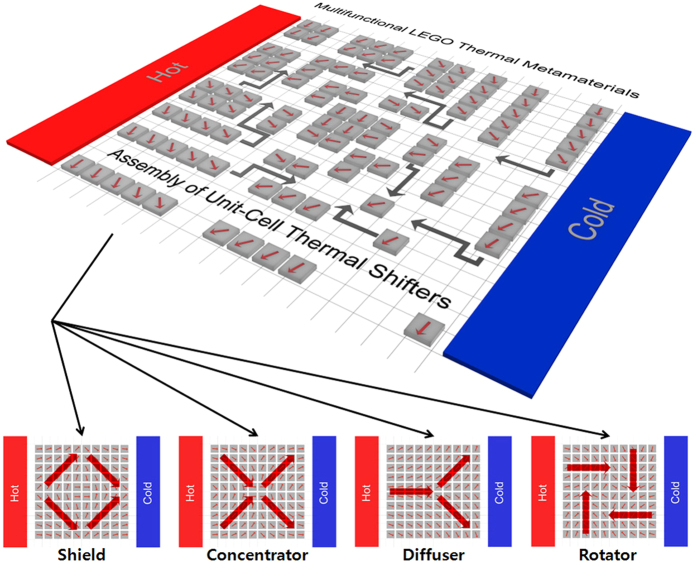
Scheme of tunable multifunctional thermal metamaterials. The designed assemblies of thermal shifters as unit-cells enables the manipulation of the local heat flux for multiple functions: thermal shifter, concentrator, diffuser, and rotator. The differently inclined thermal shifters induce the discretized heat flux lines, inspired by two-dimensional digital images. The gray arrows indicate that the specific assembly of unit-cell thermal shifters enables the certain function for local heat flux manipulation. The red arrows show that such assembled design can carry out the diverse functions, when they are differently combined.
