# Supplementary material for: Tunable Multifunctional Thermal Metamaterials: Manipulation of Local Heat Flux via Assembly of Unit-Cell Thermal Shifters
Source: Sci Rep. 2017 Jan 20;7:41000. doi: 10.1038/srep41000 (PMC5247738; doi:10.1038/srep41000)
Supplement: Supplementary Information [file srep41000-s1.pdf]

[**Supplementary Information**]

Tunable Multifunctional Thermal Metamaterials: Manipulation of Local Heat Flux via Assembly of Unit-Cell Thermal Shifters

Gwanwoo Park[1‡], Sunggu Kang[1‡], Howon Lee[2], and Wonjoon Choi[1*]

[1]School of Mechanical Engineering, Korea University, 145 Anam-ro, Seongbuk-gu, Seoul, 136-713, Republic of Korea

[2]Department of Mechanical and Aerospace Engineering, Rutgers University, 98 Brett RD, Piscataway, NJ, 08854, USA

* Author to whom any correspondence should be addressed.

E-mail: *wojchoi@korea.ac.kr*, Phone: +82 2 3290 5951, Fax: +82 2 926 9290.

‡ These authors contributed equally to this work.

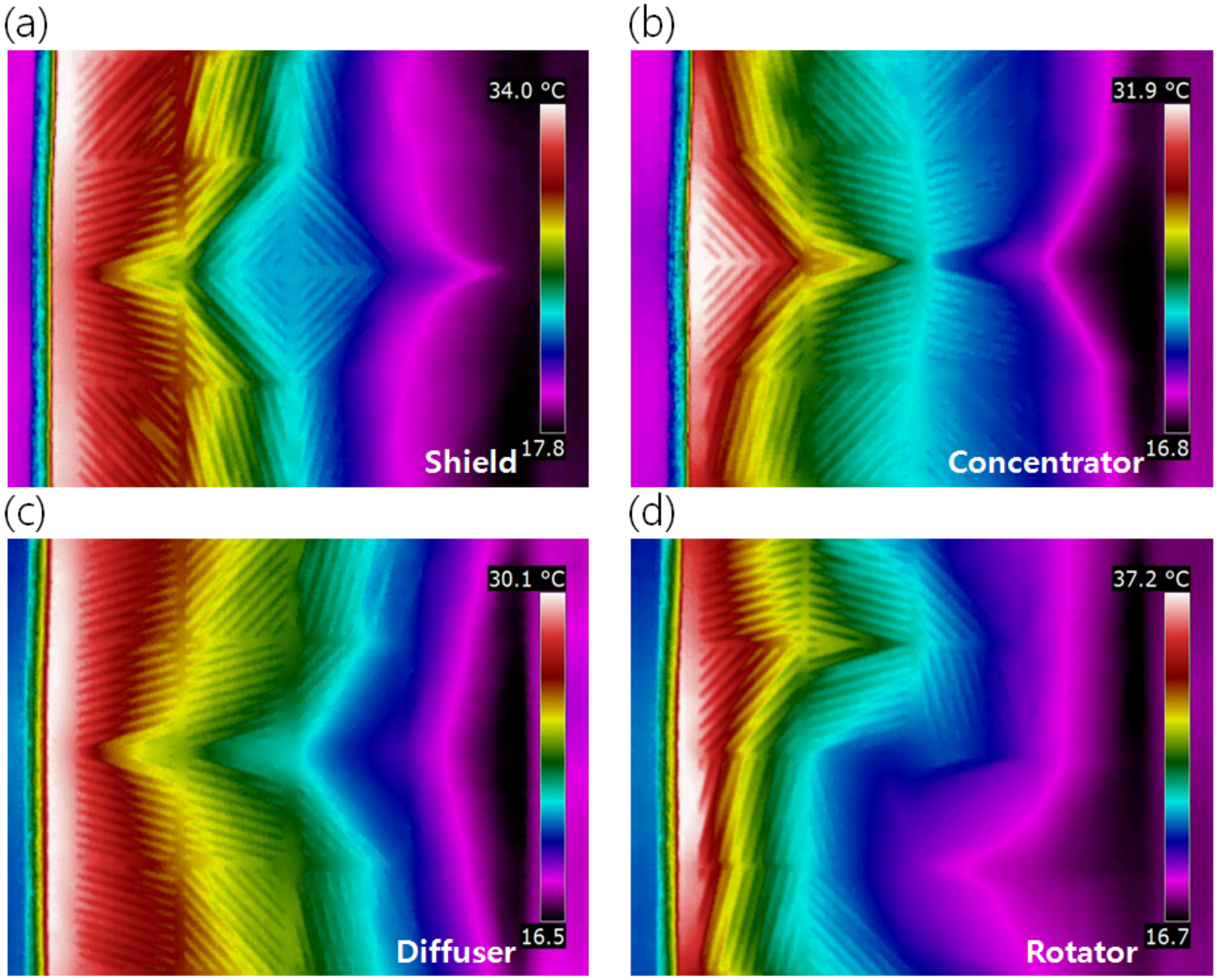

Supplementary Figure 1. Multifunctionality of thermal metamaterials via changes of assembly, made from the same 16 thermal shifters for (a) thermal shield, (b) thermal concentrator, (c) thermal diffuser and (d) thermal rotator.

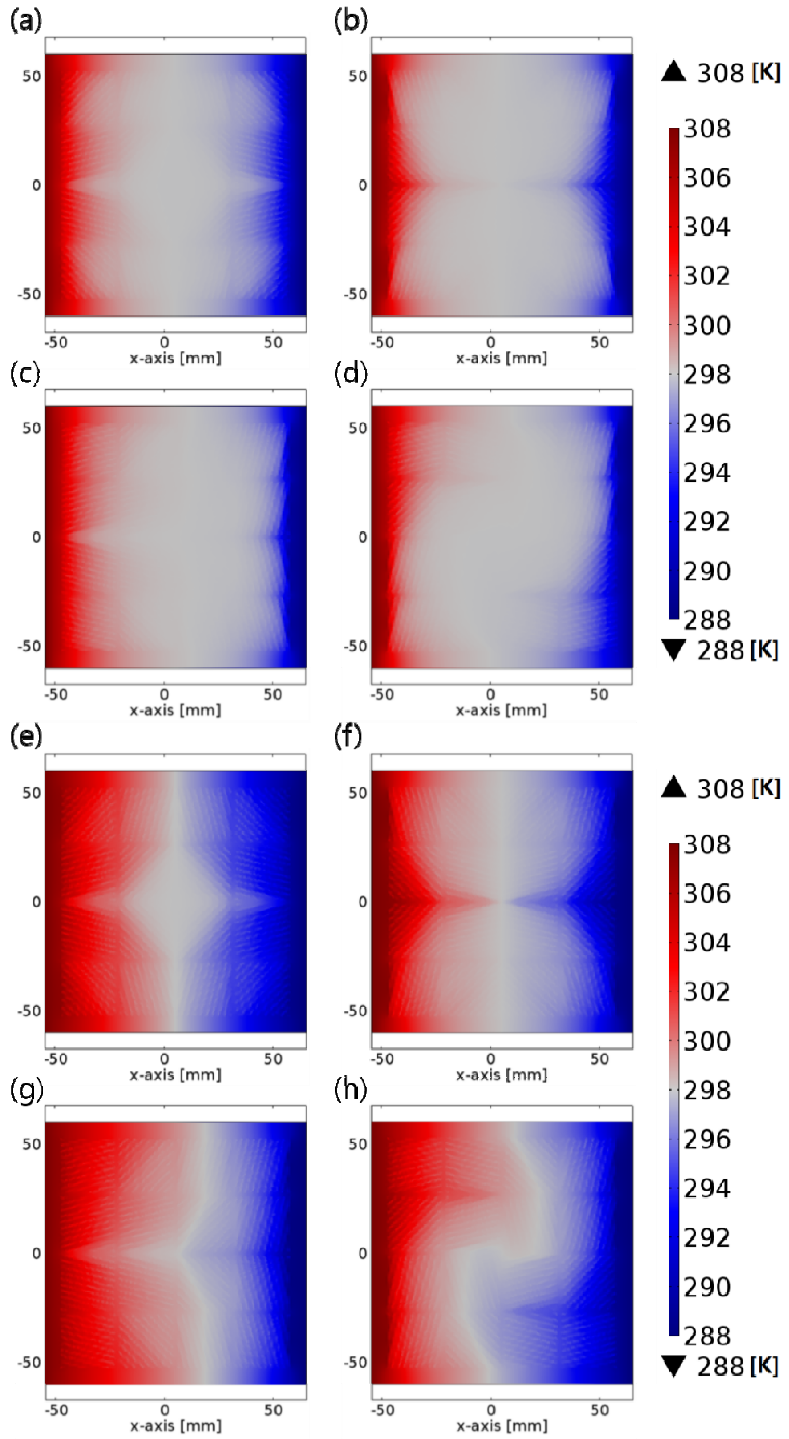

Supplementary Figure 2. Simulation of thermal metamaterial made with stainless steel (k = 16[W/m·K]) on convective condition (ambient temperature: 298K, h=10[W/m^{2}K]). (a) Thermal shield, (b) Thermal concentrator, (c) Thermal diffuser and (d) Thermal rotator. And simulation of thermal metamaterial made with 5Al bronze (k = 80[W/m·K]) on same convective condition. (e) Thermal shield, (f) Thermal concentrator, (g) Thermal diffuser and (h) Thermal rotator.

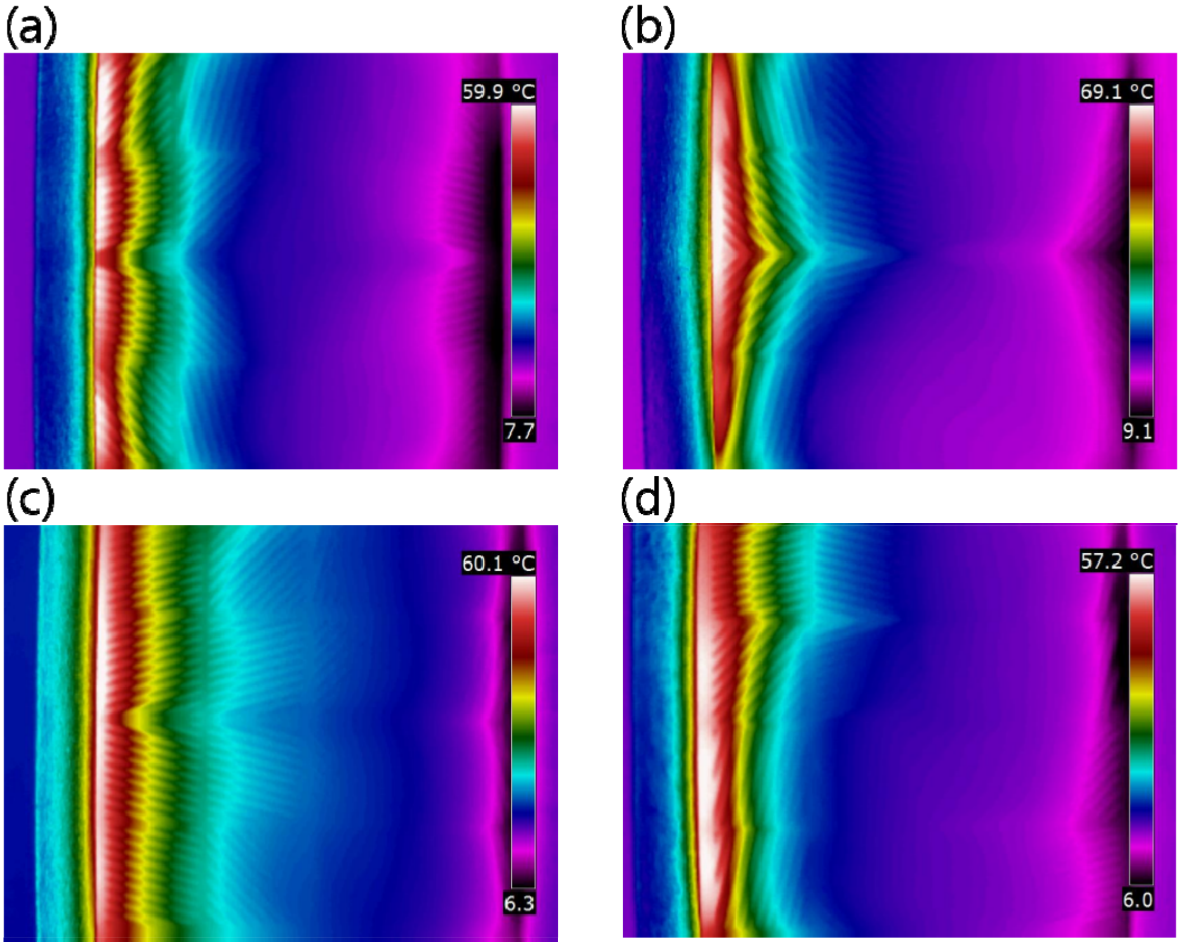

Supplementary Figure 3. Experimental temperature performance of thermal metamaterial made with stainless steel. (a) Thermal shield, (b) Thermal concentrator, (c) Thermal diffuser and (d) Thermal rotator.

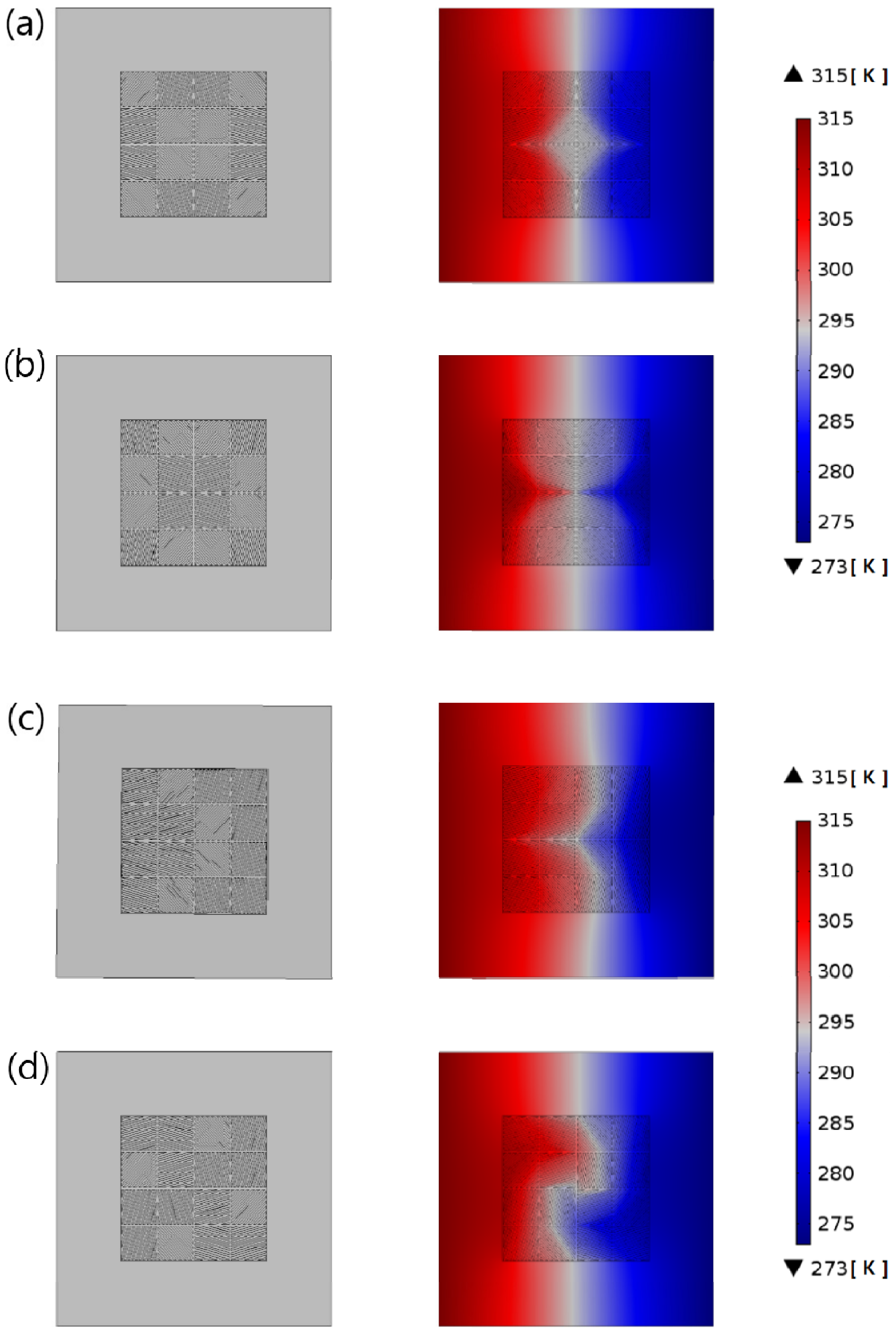

Supplementary Figure 4. Design and temperature distribution in bigger hosts (copper: k=400[W/m·K]) with 4-type thermal metamaterial, composed of copper (k=400[W/m·K]) and PDMS (k=0.2[W/m·K]). (a) Thermal shield, (b) thermal concentrator, (c) thermal diffuser and (d) thermal rotator.

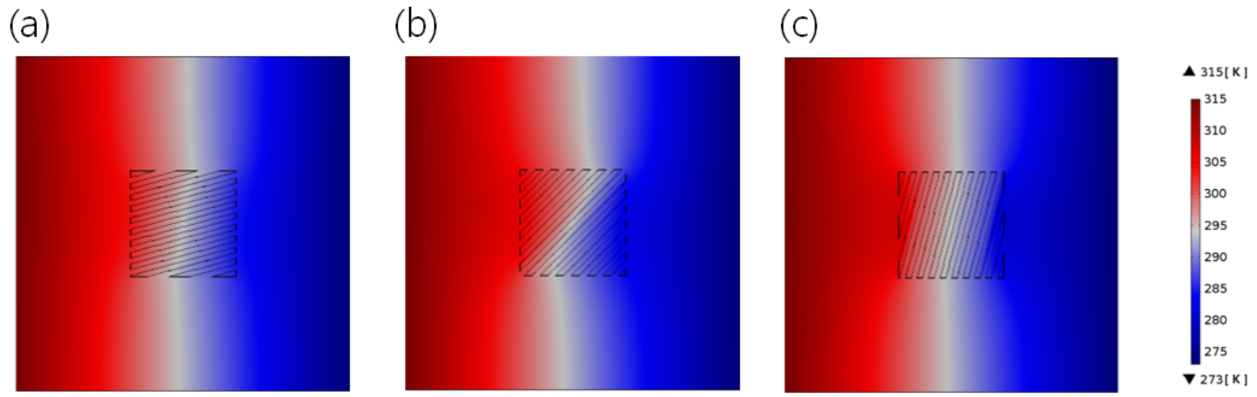

Supplementary Figure 5. Simulation results of temperature profiles for bigger hosts with thermal shifters, using FEM in (j) 15°, (k) 45°, and (l) 75°.

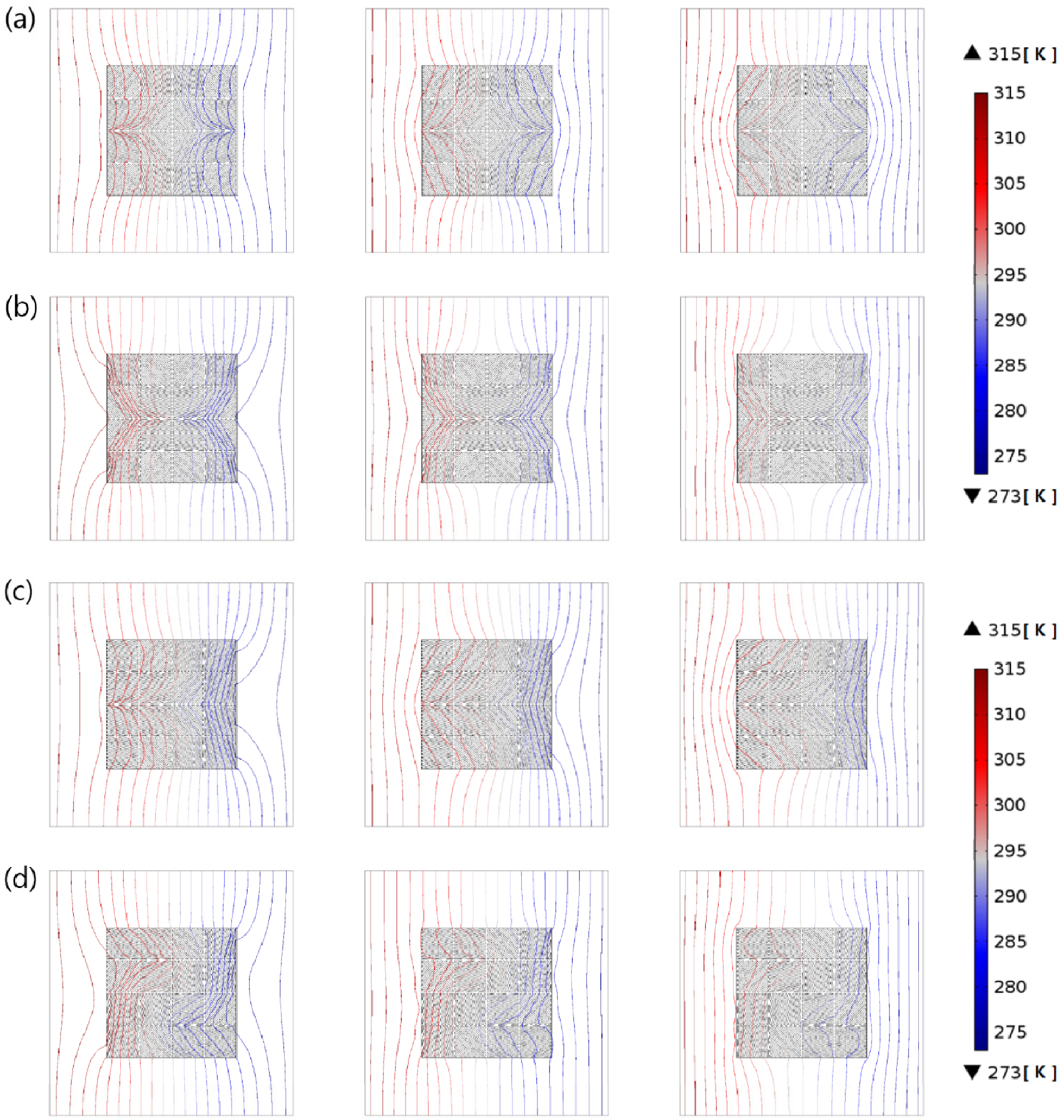

Supplementary Figure 6. Isothermal plots of bigger hosts and tunable thermal metamaterials, composed of copper (k=400[W/m·K]) and PDMS (k=0.2[W/m·K]), as thermal conductivities of bigger hosts are changed from 400[W/m·K] (left) to 100[W/m·K] (middle) to 40[W/m·K] (right). (a) Thermal shield, (b) thermal concentrator, (c) thermal diffuser and (d) thermal rotator.
